# Novel Oral Poliovirus Vaccine 2 Safety Evaluation during Nationwide Supplemental Immunization Activity, Uganda, 2022

**DOI:** 10.3201/eid3004.231361

**Published:** 2024-04

**Authors:** Farrell A. Tobolowsky, Fred Nsubuga, Zunera Gilani, Annet Kisakye, Helen Ndagije, Daniel Kyabayinze, Jane F. Gidudu

**Affiliations:** Centers for Disease Control and Prevention, Atlanta, Georgia, USA (F.A. Tobolowsky, Z. Gilani, J.F. Gidudu);; African Field Epidemiology Network, Kampala, Uganda (F. Nsubuga);; Uganda National Expanded Program on Immunizations, Kampala (F. Nsubuga, D. Kyabayinze);; World Health Organization, Kampala (A. Kisakye);; National Drug Authority, Kampala (H. Ndagije)

**Keywords:** viruses, poliovirus, vaccines, vaccine safety, Uganda

## Abstract

Given its enhanced genetic stability, novel oral poliovirus vaccine type 2 was deployed for type 2 poliovirus outbreak responses under World Health Organization Emergency Use Listing. We evaluated the safety profile of this vaccine. No safety signals were identified using a multipronged approach of passive and active surveillance.

To eliminate the risk for vaccine-related paralysis, live attenuated Sabin-strain oral poliovirus vaccine type 2 was withdrawn from routine use in a globally coordinated manner in 2016. However, because of decreased population immunity (*1*) and pathogenic reversion of vaccine virus persisting in communities or introduced during outbreak response vaccinations, circulating vaccine-derived poliovirus type 2 outbreaks have emerged across the World Health Organization (WHO) African Region, particularly during 2019‒2021 (*2*–*4*). In November 2020, to reduce the risk for new type 2 emergences, WHO granted Emergency Use Listing of a more genetically stable vaccine, novel oral poliovirus vaccine type 2 (nOPV2) (BioFarma, https://www.biofarma.co.id). Initial use began in March 2021 in a limited number of qualifying countries and subsequently expanded (*3*,*5*; https://extranet.who.int/pqweb/vaccines/polio-vaccine-novel-oral-nopv-monovalent-type-2).

Full vaccine licensure requires robust safety data and postdeployment monitoring (*6*). Although initial phase 1 and 2 clinical trials demonstrated that nOPV2 is well tolerated, those studies enrolled a small number of persons and were unlikely to detect rare adverse events; safety data were only collected actively for 7 days after vaccination (*7*,*8*). During initial use, passive and active safety surveillance implementation was not standardized, and data gaps persisted. In October 2021, after early promising safety data in large-scale use, nOPV2 rollout was expanded as recommended by the WHO Strategic Advisory Group of Experts on Immunization (*9*).

In response to 2 confirmed detections of circulating vaccine-derived poliovirus type 2 in Uganda in November 2021, a nationwide supplemental immunization activity using nOPV2 was planned for children <5 years of age. To further clarify the safety profile of nOPV2, we designed a multipronged safety evaluation using the passive surveillance system in Uganda, active hospital-based surveillance, acute flaccid paralysis (AFP) surveillance, and a cohort event monitoring system to monitor for adverse events following immunization (AEFI) and adverse events of special interest (AESI).

## The Study

We conducted a safety evaluation in Uganda after the first round of the nationwide supplemental immunization activity in January 2022. We used the country’s passive safety surveillance system to identify AEFI from any data source (e.g., telephone call, short messaging service, Open Data Kit, District Health Information System 2). We conducted active hospital-based surveillance for AESI in 18 sentinel sites. We included events that occurred within 42 days after vaccine administration (*10*). We defined AEFI as any untoward medical occurrence after immunization that did not necessarily have a causal relationship with the use of the vaccine (*11*). We defined AESI as prespecified medically significant events that have the potential to be causally associated with a vaccine product that needs to be carefully monitored and confirmed by further special studies (https://polioeradication.org/wp-content/uploads/2022/06/nOPV2-AESI-surveillance.pdf). AESI included anaphylaxis, aseptic meningitis or encephalitis, acute disseminated encephalomyelitis, Guillain-Barré syndrome/Fisher’s syndrome, myelitis/transverse myelitis, AFP, and unexplained deaths. We used the Brighton Collaboration case definitions for validation of cases (https://brightoncollaboration.us/category/pubs-tools/case-definitions) before causality assessment by an independent national AEFI committee. We recorded vaccination status by finger marking or verbal recall for events and cases.

AFP surveillance is ongoing in Uganda through passive, active, and community-based systems. AFP was defined as limb weakness in a child <15 years of age reported during and after the nOPV2 campaign to a surveillance officer or clinician; suspected cases were investigated and adjudicated by the national polio expert committee. We included AFP cases in children <5 years of age with symptom onset <42 days after nOPV2 administration.

We developed a cohort event monitoring system to prospectively monitor children for any AEFI occurring after vaccine administration. We systematically selected households in designated enumeration areas to create a nationally representative sample. We offered enrollment for all eligible children in each household; eligible children were those 0–59 months of age who had been vaccinated with the first dose of nOPV2 in the supplemental immunization activity, who would reside in the selected community for >42 days after the initial nOPV2 vaccination, who had written informed caregiver consent, who demonstrated no acute signs or symptoms at the time of vaccination, and who had a caregiver with access to a telephone. We followed vaccinated children through telephone interviews with caregivers starting on the day of vaccine administration (day 0) and on days 3, 7, 14, 28, and 42. Caregivers reported any signs or symptoms by onset date; healthcare-seeking behavior and hospitalizations were recorded. We combined and harmonized data from all 4 surveillance systems to identify any duplication.

We defined a nonspecified serious event as an event that resulted in death, required hospitalization, or resulted in persistent or major disability (*11*). For any serious event, clinicians and healthcare workers conducted detailed investigations, and the National AEFI Causality Committee of country experts used the clinical data provided to classify each event for association into 5 categories, according to global guidelines: vaccine product–related, vaccine quality–related, immunization error–related, immunization anxiety–related, indeterminate, or coincidental (*12*). We completed descriptive data analyses using Excel (Microsoft, https://www.microsoft.com) and SAS version 9.4 (SAS Institute, Inc., https://www.sas.com). The protocol received a nonresearch determination by the Uganda AIDS Support Organization Research Ethics Committee and the Uganda National Council of Science and Technology; this activity was reviewed by the Centers for Disease Control and Prevention and was conducted consistent with applicable federal law and center policy (see e.g., 45 C.F.R. part 46.102(l) (*2*), 21 C.F.R. part 56; 42 U.S.C. §241(d); 5 U.S.C. §552a; 44 U.S.C. §3501 et seq.).

In the first round of the nationwide vaccination campaign in Uganda during January 14‒21, 2022, a total of 9,768,697 doses of nOPV2 were administered to children <5 years of age. During January 14–March 11, 2022, an initial total of 1,159 AEFI were identified across all 4 safety surveillance systems (Table). Passive surveillance identified 43 AEFI; 2 (5%) of those events were serious. Through prospective active hospital-based surveillance, 8 AESI were detected; in 5 (62.5%) of those events, patients reported that they had received nOPV2 vaccination within 42 days. Among 159 AFP cases in children <5 years of age identified through the country’s active AFP surveillance, 128 (80.5%) patients reported nOPV2 receipt, and 81 (50.9%) cases were defined as serious events after investigation. Cohort event monitoring enrolled 2,213 participants (Figure); during follow-up, we found 952 AEFI, of which 22 (2%) were serious. Of the 110 conditions that were classified as serious, 6 (5.5%) were classified into the vaccine product–related reactions category, diagnosed as gastroenteritis (n = 3), acute disseminated encephalomyelitis (n = 1), encephalitis (n = 1), and acute febrile illness (n = 1).

**Table:** Adverse events identified by all surveillance systems after nOPV2 administration, Uganda, 2022*

​Event	Total	Minor AEFI†	Serious​ AEFI
​Passive surveillance	43	41	2
​​Acute flaccid paralysis surveillance	159‡	78§	81
​AESI surveillance	5	NA	5
​Cohort event monitoring	952	930	22
Total	1,159	1,049	110

*AEFI, adverse events following immunization; AESI, adverse events of special interest; nOPV2, novel oral poliovirus vaccine type 2. †AEFI are defined as any untoward medical occurrence which follows immunization that does not necessarily have a causal relationship with the usage of the vaccine. ‡Two duplicates were identified from AFP surveillance (deduplicated total = 157) that were also identified through active hospital-based surveillance. §After investigation, 78 AFP cases were downgraded by the National AEFI Committee to nonserious cases.

**Figure:**
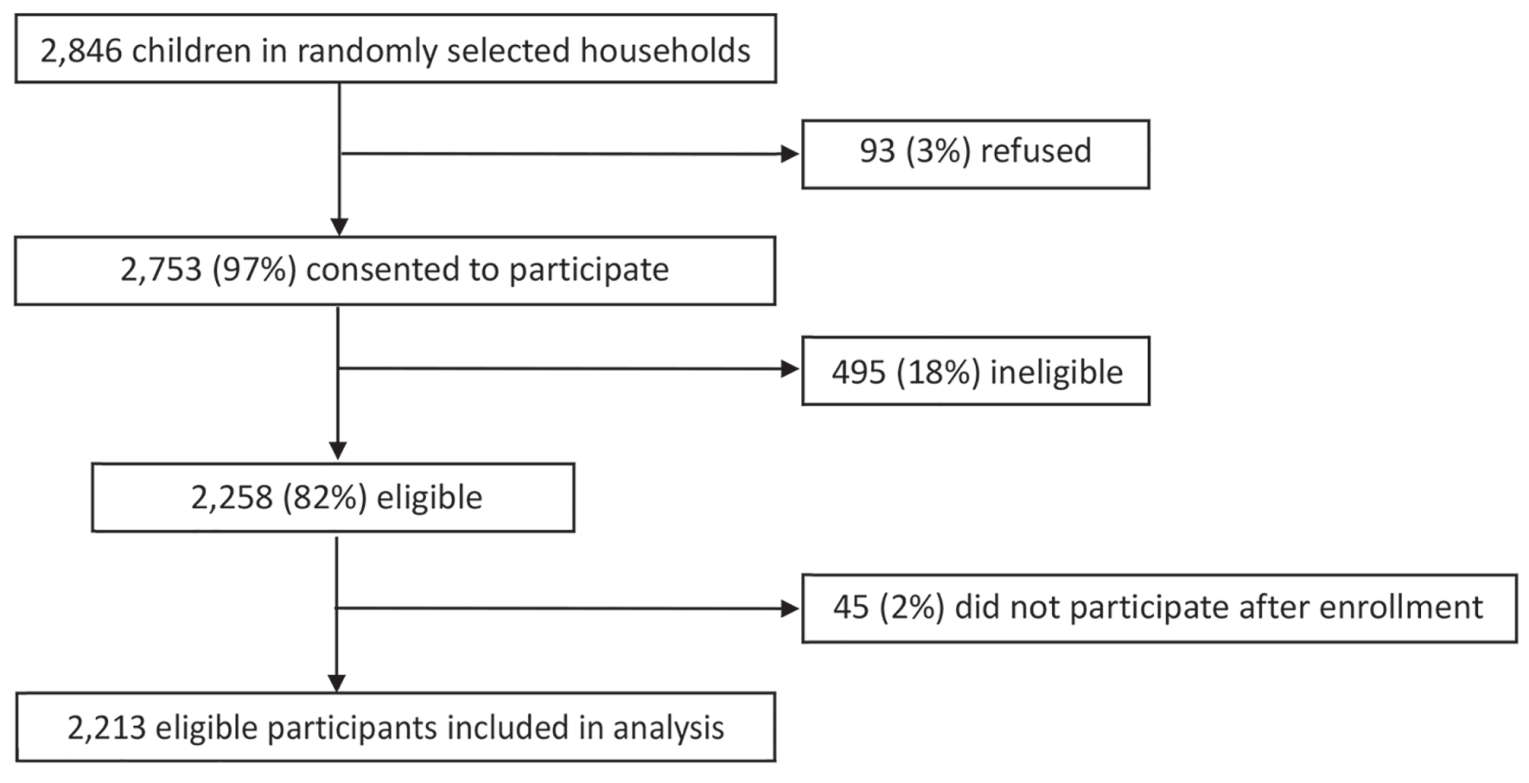
Cohort event monitoring enrollment after novel oral poliovirus vaccine type 2 administration, Uganda, 2022. Ineligible children included those who were >59 months of age, demonstrated acute signs or symptoms at the time of vaccination, were without a caretaker who had access to a phone, did not reside in the community for >42 days after vaccination, were without a caretaker staying with the child for >42 days, or did not complete enrollment, as well as any other unspecified reason.

## Conclusions

Through the multipronged surveillance system, ≈6% of serious conditions were classified by the causality committee as vaccine product–related reactions; no concerning safety signals were noted. Previous data reported from countries using nOPV2 support this finding; a similar proportion (60/1529; 4%) of events were classified as vaccine product–related reactions, and no major safety concerns have been identified outside of this evaluation in phase 3 trials (*13*–*15*).

The first limitation of this evaluation is that AEFI identified through passive surveillance were likely underreported. Conversely, soliciting events through caregivers during cohort event monitoring and active AFP surveillance might have led to overreporting. Recall bias could have occurred with reported vaccination status and in cohort event monitoring, especially with later timepoints after vaccination. Last, the COVID-19 pandemic led to delays in implementation, investigations, and timely review of conditions by the causality committee.

Our evaluation adds to the growing evidence that no safety signals are associated with nOPV2 use among persons <5 years of age. With a robust multipronged approach, safety surveillance can be strengthened for vaccines with limited safety profiles that are introduced in resource-limited settings during public health emergencies, especially while awaiting full licensure.

## References

[1] Cooper LV, Bandyopadhyay AS, Gumede N, Mach O, Mkanda P, Ndoutabé M, et al. Risk factors for the spread of vaccine-derived type 2 polioviruses after global withdrawal of trivalent oral poliovirus vaccine and the effects of outbreak responses with monovalent vaccine: a retrospective analysis of surveillance data for 51 countries in Africa. Lancet Infect Dis. 2022;22:284–94. 10.1016/S1473-3099(21)00453-9 34648733 PMC8799632

[2] Gray EJ, Cooper LV, Bandyopadhyay AS, Blake IM, Grassly NC. The origins and risk factors for serotype-2 vaccine-derived poliovirus (VDPV2) emergences in Africa during 2016–2019. J Infect Dis. 2023;228:80–8. 10.1093/infdis/jiad004 36630295 PMC10304761

[3] Alleman MM, Jorba J, Greene SA, Diop OM, Iber J, Tallis G, et al. Update on vaccine-derived poliovirus outbreaks—worldwide, July 2019–February 2020. MMWR Morb Mortal Wkly Rep. 2020;69:489–95. 10.15585/mmwr.mm6916a1 32324719 PMC7188410

[4] Alleman MM, Jorba J, Henderson E, Diop OM, Shaukat S, Traoré MA, et al. Update on vaccine-derived poliovirus outbreaks—worldwide, January 2020–June 2021. MMWR Morb Mortal Wkly Rep. 2021;70:1691–9. 10.15585/mmwr.mm7049a1 34882653 PMC8659190

[5] Bigouette JP, Henderson E, Traoré MA, Wassilak SGF, Jorba J, Mahoney F, et al. Update on vaccine-derived poliovirus outbreaks—worldwide, January 2021–December 2022. MMWR Morb Mortal Wkly Rep. 2023;72:366–71. 10.15585/mmwr.mm7214a3 37022974 PMC10078846

[6] Macklin GR, Peak C, Eisenhawer M, Kurji F, Mach O, Konz J, et al.; nOPV2 Working Group. Enabling accelerated vaccine roll-out for Public Health Emergencies of International Concern (PHEICs): Novel Oral Polio Vaccine type 2 (nOPV2) experience. Vaccine. 2023;41(Suppl 1):A122–7. 10.1016/j.vaccine.2022.02.050 35307230 PMC10109087

[7] Van Damme P, De Coster I, Bandyopadhyay AS, Revets H, Withanage K, De Smedt P, et al. The safety and immunogenicity of two novel live attenuated monovalent (serotype 2) oral poliovirus vaccines in healthy adults: a double-blind, single-centre phase 1 study. Lancet. 2019;394:148–58. 10.1016/S0140-6736(19)31279-6 31174831 PMC6626986

[8] Zaman K, Bandyopadhyay AS, Hoque M, Gast C, Yunus M, Jamil KM, et al. Evaluation of the safety, immunogenicity, and faecal shedding of novel oral polio vaccine type 2 in healthy newborn infants in Bangladesh: a randomised, controlled, phase 2 clinical trial. Lancet. 2023;401:131–9. 10.1016/S0140-6736(22)02397-2 36495882 PMC9860215

[9] Global Polio Eradication Initiative. Independent experts advise move to next use phase for novel oral polio vaccine type 2. 2021 Oct 11 [cited 2023 Mar 7]. https://polioeradication.org/news-post/independent-experts-advise-transition-to-next-use-phase-for-novel-oral-polio-vaccine-type-2-nopv2/

[10] Rowhani-Rahbar A, Klein NP, Dekker CL, Edwards KM, Marchant CD, Vellozzi C, et al.; Risk Interval Working Group of the Clinical Immunization Safety Assessment Network. Biologically plausible and evidence-based risk intervals in immunization safety research. Vaccine. 2012;31:271–7. 10.1016/j.vaccine.2012.07.024 22835735

[11] World Health Organization. Adverse events following immunization (AEFI) [cited 2023 May 26]. https://iris.who.int/bitstream/handle/10665/191391/a87773_eng.pdf

[12] World Health Organization. Causality assessment of an adverse event following immunization (‎AEFI)‎: user manual for the revised WHO classification, 2nd ed., 2019 update [cited 2023 Apr 10]. https://iris.who.int/bitstream/handle/10665/340802/9789241516990-eng.pdf

[13] Global Polio Eradication Initiative. GACVS (Global Advisory Committee on Vaccine Safety) sub-committee on novel type 2 oral poliovirus vaccine (nOPV2) safety assessment of nOPV2 safety data. 2023 Jan 24 [cited 2023 Oct 1]. https://polioeradication.org/wp-content/uploads/2023/03/GACVS-nOPV2-committee-meeting-20230124.pdf

[14] World Health Organization. Safety profile of nOPV2 vaccine [cited 2024 Jan 10]. https://www.who.int/groups/global-advisory-committee-on-vaccine-safety/topics/poliovirus-vaccines

[15] Rivera Mejía L, Peña Méndez L, Bandyopadhyay AS, Gast C, Mazara S, Rodriguez K, et al. Safety and immunogenicity of shorter interval schedules of the novel oral poliovirus vaccine type 2 in infants: a phase 3, randomised, controlled, non-inferiority study in the Dominican Republic. Lancet Infect Dis. 2023;S1473-3099(23)00519-4. 10.1016/S1473-3099(23)00519-4 PMC10881405 38109921

